# GC-compositional strand bias around transcription start sites in plants and fungi

**DOI:** 10.1186/1471-2164-6-26

**Published:** 2005-02-28

**Authors:** Shigeo Fujimori, Takanori Washio, Masaru Tomita

**Affiliations:** 1 Institute for Advanced Biosciences, Keio University, Tsuruoka, Yamagata 997-0035, Japan; 2 Graduate School of Media and Governance, Keio University, Fujisawa, Kanagawa 252-8520, Japan; 3 Graduate School of Information Science, Nara Institute of Science and Technology, Ikoma, Nara 630-0192, Japan; 4 Department of Environmental Information, Keio University, Fujisawa, Kanagawa 252-8520, Japan

## Abstract

**Background:**

A GC-compositional strand bias or GC-skew (=(C-G)/(C+G)), where C and G denote the numbers of cytosine and guanine residues, was recently reported near the transcription start sites (TSS) of *Arabidopsis *genes. However, it is unclear whether other eukaryotic species have equally prominent GC-skews, and the biological meaning of this trait remains unknown.

**Results:**

Our study confirmed a significant GC-skew (C > G) in the TSS of *Oryza sativa *(rice) genes. The full-length cDNAs and genomic sequences from *Arabidopsis *and rice were compared using statistical analyses. Despite marked differences in the G+C content around the TSS in the two plants, the degrees of bias were almost identical. Although slight GC-skew peaks, including opposite skews (C < G), were detected around the TSS of genes in human and *Drosophila*, they were qualitatively and quantitatively different from those identified in plants. However, plant-like GC-skew in regions upstream of the translation initiation sites (TIS) in some fungi was identified following analyses of the expressed sequence tags and/or genomic sequences from other species. On the basis of our dataset, we estimated that >70 and 68% of *Arabidopsis *and rice genes, respectively, had a strong GC-skew (>0.33) in a 100-bp window (that is, the number of C residues was more than double the number of G residues in a +/-100-bp window around the TSS). The mean GC-skew value in the TSS of highly-expressed genes in *Arabidopsis *was significantly greater than that of genes with low expression levels. Many of the GC-skew peaks were preferentially located near the TSS, so we examined the potential value of GC-skew as an index for TSS identification. Our results confirm that the GC-skew can be used to assist the TSS prediction in plant genomes.

**Conclusion:**

The GC-skew (C > G) around the TSS is strictly conserved between monocot and eudicot plants (ie. angiosperms in general), and a similar skew has been observed in some fungi. Highly-expressed *Arabidopsis *genes had overall a more marked GC-skew in the TSS compared to genes with low expression levels. We therefore propose that the GC-skew around the TSS in some plants and fungi is related to transcription. It might be caused by mutations during transcription initiation or the frequent use of transcription factor-biding sites having a strand preference. In addition, GC-skew is a good candidate index for TSS prediction in plant genomes, where there is a lack of correlation among CpG islands and genes.

## Background

A prominent GC-compositional strand bias or GC-skew (=(C-G)/(C+G)), where C and G denote the numbers of cytosine and guanine residues, was reported recently around the transcription start sites (TSS) of *Arabidopsis *genes [1]. It is well known that GC-skews occur bi-directionally in circular bacterial genomes along the direction of replication, and that GC-skew is an effective index for predicting the replication origin in some bacteria [2,3]. In this case the numbers of G and T (thymine) residues in the leading strand of these genomes exceed those of C and A (adenine). Several models have been proposed to explain this bias [4]. A similar strand bias related to the direction of replication has also been observed in human mitochondrial genomes [5]. Also, mammalian and enterobacterial genomes have been reported to show a strand bias associated with transcribed regions [6-8]. An excess of G+T over A+C was observed in mammals within the sense strand of genes. A transcription-coupled DNA-repair system might be involved in this bias [9]. However, existing models cannot explain the excess of C over G in the sense strand around the TSS in *Arabidopsis*. Although slight GC-skews (regardless of the direction) were reported recently around the TSS in metazoans [10,11], it remains unclear whether the strong GC-skew of *Arabidopsis *is similar to that observed in metazoans.

Although large amounts of genomic and full-length cDNA sequence data from plants are now publicly available, knowledge of the promoters and TSS in plants is still limited compared to mammals, such as human and mouse. It has been reported that the CpG island [12] is the most effective index for predicting the promoter regions or TSS in mammals[13,14]. However, the CpG islands are not specifically located in the promoter regions in *Arabidopsis*, so they cannot be used for the prediction of TSS or promoters [15]. Identifying another, more suitable, index for the prediction of plant-specific TSS has therefore become a priority.

Answers are required for two key issues. First, which eukaryotic species, phyla or kingdoms have *Arabidopsis*-like GC-skews around the TSS? Second, what is the biological significance of these regions? In the present study, we used sequences from various animal, fungus, protist and plant species to conduct comparative analyses. We explored the potential value of GC-skew as an index for TSS prediction in plants. Finally, we considered the biological meaning of the GC-skew around the TSS of plant genes.

## Results

### GC-skew around the TSS of plant genes

**Figure 1:**
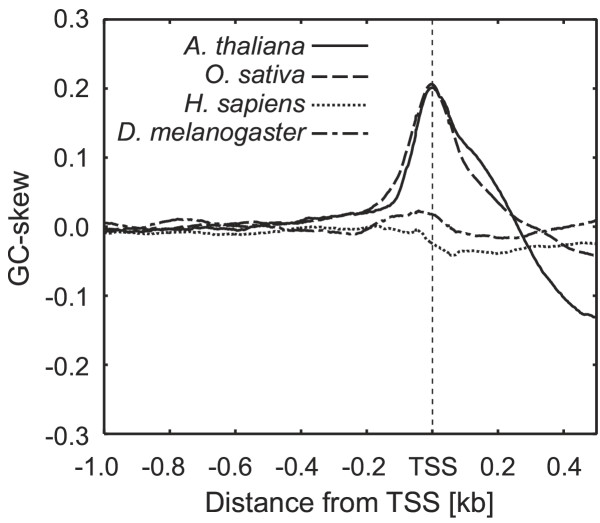
**GC-skew in up- and downstream regions of the TSS. **The up- and downstream regions of the TSS, which were 1.0 and 0.5-kb long, were analyzed using data from four species: 7,708 loci for *Arabidopsis*, 14,868 loci for rice, 14,053 loci for human and 8,344 loci for *Drosophila*. The graph shows the mean GC-skew values calculated for sequences of the four species using the sliding-window technique (window size = 100 bp; shift size = 1 bp).

The shift in GC-skew values around TSS was assessed by calculating the GC-skew for regions between 1.0-kb upstream and 0.5-kb downstream of the TSS in *Arabidopsis *(a dicotyledonous plant), rice (a monocotyledonous plant), human and *Drosophila*. Full-length cDNAs and genomic sequences were exploited from the species with available TSS data. The sliding-window method was used, and a value for the GC-skew was computed at the central position for each 100 bp. The average of the GC-skew values at each position was calculated for all of the genes. The results confirmed that the mean GC-skew spectra of both *Arabidopsis *and rice peaked at the same position as the TSS (Fig. 1). The mean GC-skew values were approximately zero in regions upstream (<-0.2 kb) from the TSS, as the numbers of C and G residues were equal in both plant species. The values increased from the proximal region (-0.2 to -0.1 kb) and peaked at the TSS. Downstream from the TSS, the mean GC-skew values were inversely low. Although a small GC-skew was detected around the TSS in *Drosophila *and an opposite skew (C < G) was observed in human, these were less prominent when compared to those in plants. Our findings for *Drosophila *and human were consistent with previous data [10,11], indicating that this is a plant- or angiosperm-specific phenomenon. Intriguingly, the mean GC-skew values at the TSS were identical in *Arabidopsis *and rice genes, which were 0.20 (standard deviation = 0.30) and 0.20 (standard deviation = 0.29), respectively. However, it should also be noted that the mean values for the G+C content in the TSS region (-50 to +50 bp) in *Arabidopsis *and rice were significantly different: 37 and 53%, respectively. In contrast, the G:C ratios at the TSS in *Arabidopsis *and rice were identical, despite the considerable difference in G+C content.

We also determined the frequencies of the four nucleotides in the regions up- and downstream of the TSS in plants (see Fig. S1 in Additional file 1). High C-residue frequencies were observed in both *Arabidopsis *and rice (approximately 50 to 100 bp from the TSS). In contrast, G-residue frequencies decreased slightly around the TSS (+/-10 bp). These findings suggest that the peaks of GC-skew values observed near the plant TSS were caused by an increased frequency of C residues and a slight reduction in the G-residue frequency. No significant biases were observed in the frequencies of A and T residues, indicating a lack of AT-skew at the TSS (See Fig. S2 in Additional file 1).

**Figure 2:**
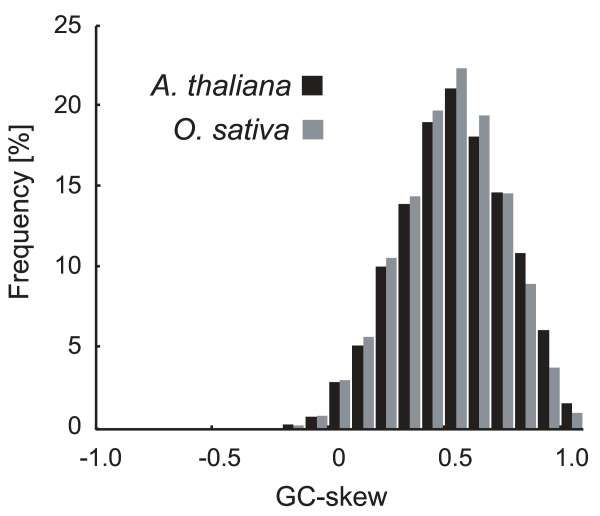
**Distribution of maximum GC-skew values around the TSS in *Arabidopsis *and rice genes. **The graph illustrates the distribution of maximum GC-skew values within 100-bp up- and downstream of the TSS in *Arabidopsis *and rice. The GC-skew values were computed using the sliding-window technique (window size = 100 bp; shift size = 1 bp). The numbers of sequences analyzed were 7,708 for *Arabidopsis *and 14,868 for rice.

The number of plant genes with a strong GC-skew in proximal regions of the TSS, was calculated by assessing the distributions of GC-skew values in regions +/-100 bp of the TSS. In order to take into account the wobble of the TSS, and to avoid experimental or mapping artifacts, only the maximum GC-skew values from these regions were used. The distributions of the maximum GC-skew values in the proximal regions of the TSS were similar in both plant species (Fig. 2). Over 70% of *Arabidopsis *genes and over 68% of rice genes showed a strong GC-skew (>0.33) near the TSS (that is, the number of C residues being more than double that of the G residues).

Our results suggest that many plant (*Arabidopsis *and rice) genes have strong GC-skews around the TSS. Furthermore, this characteristic is common among both monocot and eudicot plants. In contrast, the GC-skews (including the C < G skew) that were observed in human and *Drosophila *were qualitatively and quantitatively different from those identified in plants.

### GC-skew in eukaryotes

The existence of GC-skew peaks around the TSS in various eukaryotes was examined by calculating GC-skew values 100-bp downstream of the 5' -ends of virtually assembled transcripts [16,17]. Although these did not represent the actual TSS, we were able to approximate the GC-skew values in the downstream regions. Table 1 shows the mean GC-skew values in the downstream regions of the TSS for several species. A prominent GC-skew (an excess of C residues) was confirmed in the 5' -ends of the transcripts in seven out of the nine plant species examined, and in five out of seven species of fungi. Although opposite skews (C < G) were observed in several protist and animal species, no significant excess of C residues was detected in any of the 10 animal species or the 11 protist species analyzed.

**Table 1:** GC-skew in various eukaryotes

Group	Species	GC-skew	Mean	Std. Dev.	No. of sequences
Plant	*Sorghum bicolor*	++	0.126	0.278	4,482
	*Oryza sativa*	++	0.118	0.304	18,676
	*Triticum aestivum*	+	0.095	0.255	13,133
	*Arabidopsis thaliana*	+	0.094	0.303	18,714
	*Gossypium*	+	0.092	0.304	1,561
	*Zea mays*	+	0.075	0.247	7,904
	*Glycine max*	+	0.050	0.311	6,162
	*Chlamydomonas reinhardtii*		0.032	0.184	3,343
	*Pinus luchuensis*		-0.045	0.227	3,896

Fungus	*Filobasidiella neoformans*	++	0.222	0.341	243
	*Neurospora crassa*	++	0.184	0.276	2,763
	*Coccidioides immitis*	++	0.174	0.317	52
	*Aspergillus nidulans*	++	0.139	0.238	254
	*Magnaporthe grisea*	++	0.126	0.224	2,799
	*Saccharomyes cerevisiae*		-0.012	0.188	2,642
	*Schizosaccharomyces pombe*		-0.032	0.189	1,489

Protist	*Eimeria tenella*		0.046	0.195	300
	*Tetrahymena thermophila*		0.040	0.249	171
	*Trichomonas vaginalis*		0.037	0.167	47
	*Dictyostelium discoideum*		0.015	0.328	2,032
	*Neospora caninum*		-0.004	0.169	636
	*Toxoplasma gondii*		-0.019	0.184	1,328
	*Sarcocystis neurona*		-0.035	0.183	91
	*Trypanosoma brucei*	-	-0.080	0.230	231
	*Plasmodium berghei*	--	-0.102	0.308	86
	*Cryptosporidium parvum*	--	-0.124	0.259	70
	*Plasmodium falciparum*	--	-0.191	0.354	1,905

Animal	*Caenorhabditis elegans*		0.008	0.194	8,848
	*Drosophila melanogaster*		-0.011	0.170	14,310
	*Amblyomma variegatum*		-0.015	0.152	77
	*Ictalurus punctatus*		-0.017	0.216	382
	*Rattus norvegicus*		-0.023	0.193	12,594
	*Danio rerio*		-0.029	0.198	9,350
	*Homo sapiens*		-0.045	0.213	53,459
	*Mus musculus*		-0.045	0.217	50,029
	*Xenopus laevis*	-	-0.064	0.212	13,444
	*Schistosoma mansoni*	-	-0.088	0.230	195

The mean values and standard deviations (Std. Dev.) of the GC-skew values 100-bp downstream of the 5' -end were calculated in virtually assembled transcripts of nine plant species, seven species of fungus, 11 protist species and 10 animal species, which were downloaded from [16, 17]. The symbols + and ++ denote the predominance of C: ++ (≥0.10) and + (≥0.05). The symbols - and -- denote the predominance of G: -- (≤-0.10) and - (≤-0.05).

**Figure 3:**
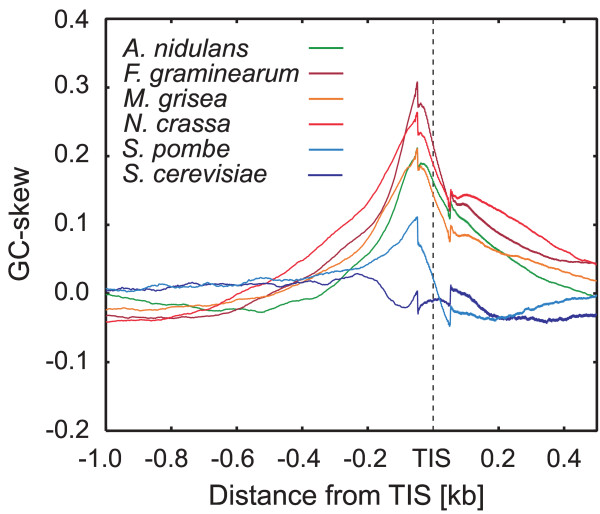
**GC-skew around TIS in fungal genes. **The mean GC-skew values in regions 1.0-kb upstream and 0.5-kb downstream of TIS in the genomes of six species of fungi were calculated using the sliding-window technique (window size = 100 bp; shift size = 1 bp) for 9,432, 11,407, 10,054, 9,872, 5,825 and 4,305 loci in *A. nidulans*, *F. graminearum*, *M. grisea*, *N. crassa*, *S. cerevisiae *and *S. pombe*, respectively.

We determined whether GC-skew peaks were actually present in the TSS of fungal genes, by investigating the regions around the translation initiation sites (TIS) of fungal genomic sequences. Genomic sequence data and information on open reading frames (ORFs; including predicted ones) are publicly available for some fungi, although there is insufficient information about the TSS. Nevertheless, it was possible to estimate the tendency towards GC-skew around the TSS by analyzing sequences both up- and downstream of the TIS in the available genomic sequences. Figure 3 shows the mean GC-skew values in regions between 1.0-kb upstream and 0.5-kb downstream of the TIS in fungal species: *Aspergillus nidulans*, *Fusarium graminearum, Magnaporthe grisea*, *Neurospora crassa*, *Saccharomyces cerevisiae *and *Schizosaccharomyces pombe*. GC-skew peaks similar to those of plants were observed in 50- to 100-bp upstream regions of the TIS in all of these species, with the exception of *S. cerevisiae*.

### Correlation between the two nucleotide frequencies in plants

**Figure 4:**
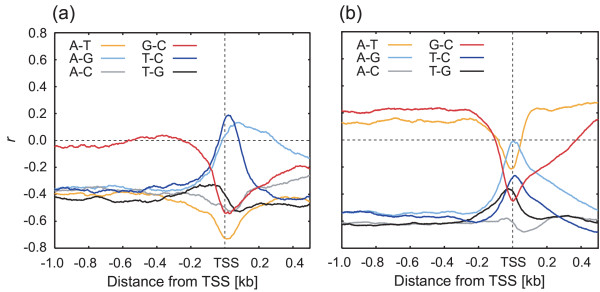
**Correlation between the two nucleotide frequencies in plants. **The figure illustrates the correlation coefficients (*r*) between the two nucleotide frequencies (A-T, A-G, A-C, G-C, T-C and T-G) at each position around the TSS in (**a**) *Arabidopsis *and (**b**) rice genes. Each nucleotide frequency at a particular position was defined as the frequency of the nucleotide in a 100-bp window at that position. Correlation coefficients were calculated using these frequencies (see Methods section). The numbers of sequences analyzed were 7,708 for *Arabidopsis *and 14,868 for rice.

A possible mechanism causing the GC-skew around the TSS is nucleotide substitution, raising the question of what kind of substitution could be responsible. Correlations between the two nucleotide frequencies in regions both up- and downstream of the TSS were expected to indicate the answer (see Fig. 4 for *Arabidopsis *and rice, and Fig. S3 in Additional file 1 for human and *Drosophila*). If the substitution rate between two specific nucleotides in one region is higher than that in other regions, a larger negative correlation would be expected between the two nucleotide frequencies, compared to other regions. In both plant species, the correlation coefficient (*r*) of A-T and G-C decreased dramatically around the TSS: the values were -0.4 to -0.7, and 0.0 to -0.5, in *Arabidopsis *(Fig. 4(a)), respectively; and 0.18 to -0.2, and 0.2 to -0.4, in rice (Fig. 4(b)), respectively. In contrast, the *r *values of A-G and T-C increased significantly around the TSS: values were -0.4 to 0.1, and -0.4 to 0.2, in *Arabidopsis *(Fig. 4(a)), and -0.5 to 0.0, and -0.6 to -0.3, in rice (Fig. 4(b)).

### GC-skew and gene-expression level

In order to examine the relationship between GC-skew at the TSS and gene expression in plants, we conducted a statistical test using serial analysis of gene expression (SAGE) data for *Arabidopsis *(10-day-old seedlings) [18]. The mean GC-skew value in the TSS of highly-expressed genes (see Methods section for details) was significantly higher (*P *= 0.0003, paired t-test) than in genes with low expression in *Arabidopsis*: the mean values were 0.25 (standard deviation = 0.31) and 0.19 (standard deviation = 0.31).

### Potential value of GC-skew as an index for TSS prediction

Many of the GC-skew peaks were preferentially located near the TSS, therefore we assessed the potential value of GC-skew as a predictive index for TSS in plants. Sequences 1-kb upstream of the predicted ORF start positions in plant genomic sequences were used in the following analysis.

First, the GC-skew values were computed using the sliding-window technique, in which one window and the shift size were set to 100 and 1 bp, respectively. GC-skew peaks satisfying a particular cut-off value were identified as primitive TSS candidates from the noisy GC-skew spectrum using the Savitzky-Golay (S-G) filter [19], which simultaneously smoothes and differentiates. Next, TSS candidates that were located within 50 bp of another candidate were considered to be identical and were merged into the position with the highest GC-skew. TSS prediction was validated by counting as true positives (*TP*), the candidates that were located within 100-bp up- or downstream of the actual TSS. If more than two candidates coexisted in the appropriate region, they were regarded as one *TP*.

**Figure 5:**
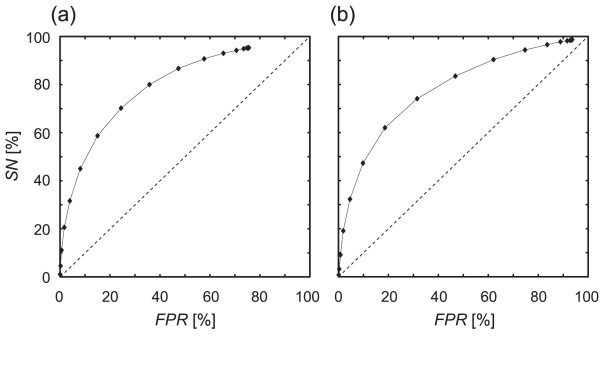
**ROC curves for predictions by GC-skew peak. **ROC curves for (**a**) *Arabidopsis *and (**b**) rice. The areas under the curves (AUCs) for ROC curves were 0.80 for *Arabidopsis *and 0.78 for rice. The diagonal lines in the two graphs correspond to the ROC curve produced by random prediction.

Using this method and the criteria described above, we validated the predictive performance of GC-skew under cut-off values ranging from -0.9 to 0.9 (Table 2). The specificity (*SP = TP/(TP+FP)*) ranged from 14 to 87%, and the sensitivity (*SN = TP/(TP+FN)*) ranged from 1 to 95% in *Arabidopsis*. The *SP *in rice ranged from 12 to 56%, and the *SN *ranged from 1 to 99%. The false-positive rate (*FPR = FP/*(*TN+FP*)) varied with the cut-off values in a similar manner to *SN*. The difference between our results and random cases was clarified using a receiver-operating characteristic (ROC) curve (Fig. 5). This is a plot of *FPR *versus *SN*, with each cut-off value corresponding to a point on the curve. Good ROC curves lie closer to the top left-hand corner, whereas the random cases are represented as a diagonal line (defined by *FPR = SN*). Predictions made using the GC-skew appeared to differ from the random cases, and lay closer to the top left-hand corner in both *Arabidopsis *and rice. In addition, the correlation coefficient (*φ*; see Methods section for details) corresponding to each GC-skew cut-off value was calculated in order to compare their predictive performances. The GC-skew value that maximized *φ *was 0.4 in both *Arabidopsis *and rice (*SP *= 45%, *SN *= 41% and *FPR *= 8%, and *SP *= 47%, *SN *= 38% and *FPR *= 10%, respectively).

**Table 2:** TSS prediction results for a stepwise increase of cut-off values

*Arabidopsis*					Rice			
GC-skew cutoff	*SN (%)*	*SP *(%)	*FPR *(%)	*φ*	*SN (%)*	*SP *(%)	*FPR *(%)	*φ*

-0.9	95.3	13.6	24.4	0.149	98.7	11.6	6.4	0.068
-0.8	95.3	13.6	24.5	0.149	98.7	11.6	6.4	0.068
-0.7	95.3	13.6	24.5	0.150	98.6	11.7	6.5	0.068
-0.6	95.2	13.7	24.7	0.150	98.6	11.7	6.7	0.069
-0.5	95.2	13.7	25.2	0.153	98.4	11.7	7.2	0.072
-0.4	94.9	13.9	26.5	0.157	98.2	11.8	8.4	0.078
-0.3	94.2	14.3	29.4	0.168	97.8	12.1	11.1	0.092
-0.2	93.0	15.1	34.6	0.187	96.6	12.6	16.4	0.115
-0.1	90.7	16.4	42.3	0.213	94.4	13.6	25.3	0.147
0.0	86.7	18.6	52.6	0.247	90.4	15.4	37.9	0.186
0.1	80.0	21.8	64.2	0.282	83.5	18.2	53.2	0.230
0.2	70.2	26.4	75.6	0.315	74.1	22.7	68.5	0.279
0.3	58.7	32.9	85.0	0.345	62.0	29.4	81.4	0.322
0.4	45.0	40.9	91.9	0.354*	47.3	37.7	90.2	0.340*
0.5	31.6	50.5	96.1	0.343	32.3	46.7	95.4	0.327
0.6	20.6	60.6	98.3	0.312	19.1	55.3	98.1	0.281
0.7	11.1	70.2	99.4	0.251	9.1	60.2	99.2	0.204
0.8	4.6	77.4	99.8	0.171	3.2	59.4	99.7	0.119
0.9	1.1	86.9	100.0	0.089	0.8	56.3	99.9	0.057

Only the TSS of genes in which a TIS was defined within the 1.0-kb downstream region are included. Sequence data from 1.0-kb upstream of the TIS (6,850 sequences for *Arabidopsis *and 13,111 sequences for rice) were used for the TSS prediction. The asterisks denote maximum *φ*.

## Discussion

This study confirmed the presence of significant GC-skews (C > G) around the TSS (or upstream regions of the TIS) of genes in some species of plants and fungi. In contrast, our analysis revealed no significant excess of C residues in either animals or protists. However, an opposite GC-skew (C < G) has been reported previously in several animal species (*Mus musculus, Rattus norvegicus, Fugu rubripes, Danio rerio *and human) [10]. Although small skews were detected for these species in our present analysis (Table 1; Fig. 1), they were not significant compared to those observed in plants. Aerts *et al*. [10] reported a GC-skew (C > G) close to the TSS in two nematode species (*Caenorhabditis elegans *and *Caenorhabditis briggsae*) however, no significant GC-skew was detected in *C. elegans *in our analysis. This inconsistency was probably due to the fact that our analysis targeted only regions downstream of the TSS in *C. elegans *(thus, regions upstream were not examined); alternatively, the skew might have been too small to be detected using our method. In either case, it is difficult to compare GC-skews between nematodes and plants, since trans-splicing at the 5' -ends of genes has been reported in nematodes [20,21], as pointed out by Aerts *et al *[10]. As noted above, the GC-skews near the TSS in plants and fungi differed from those of other species (or kingdoms) in both quality and quantity. To our knowledge, this is the first report to describe the prominent GC-skew (C > G) around the TSS specific to plants and fungi.

We propose two possible explanations for the GC-skew peaks found close to the TSS. First, regulatory elements, such as transcription factor-biding sites (TFBS), which are present in regions both up- and downstream of the TSS, might contribute to (or even cause) this phenomenon. Moreover, some TFBS have a strand preference (see for example [22]). Therefore, if these types of TFBS are preferentially located around the TSS of plant and fungal genes, they might influence the local GC-compositional strand bias. Second, the GC-skew might be involved in transcription-coupled events, such as transcription-associated mutational asymmetry [6-9]. Tatarinova and colleagues [1] mentioned such transcription-associated mutational asymmetry and suggested that the GC-skew around the TSS of genes might be caused by the substitution of C residues with T residues, due to C deamination in the template strand. However, this hypothesis cannot fully explain all of the findings of our present study. If the C-to-T transition occurred preferentially around the TSS in the template strand, an AT-skew would also be expected in these regions. However, no significant AT-skew (see Fig. S2 in Additional file 1) was observed at the TSS of either *Arabidopsis *or rice genes, indicating that the C-to-T transition was not the main cause of the GC-skew. Furthermore, in our analyses, significant changes in the correlation coefficient (decreased A-T/G-C and increased A-G/T-C) were observed in both *Arabidopsis *and rice (Fig. 4). Assuming that the GC skew is caused by mutations during transcription initiation, changes in the correlation coefficient might be interpreted as an increase in the transversion ratio around the TSS. The increased negative correlation between C and G, coupled with the high GC-skew value in the same region, led us to speculate that G-to-C transversion occurred at relatively high rates in this region in plants and fungi, but not in animals and protists. If mutations that yield a GC-skew occur mainly around the TSS of single-stranded DNA, highly-expressed genes would be expected to have a high GC-skew around the TSS. In fact, the mean GC-skew value in the TSS of highly-expressed genes was significantly higher than in genes with low expression in *Arabidopsis*. This indicates that the GC-skew is associated with the level of gene expression, at least in *Arabidopsis*. Strand-specific mutational rates are believed to be a by-product of transcription-coupled DNA repair in mammals [8], therefore the GC-skew observed around the TSS of plant genes might result from the plant-specific DNA lesion and repair system. Alternatively, if the GC-skew was caused by the higher frequency of strand-specific TFBS, the higher mean GC-skew value in highly-expressed genes might be interpreted as a greater effect of strand-specific TFBS for the transcription efficiency. In either strand-specific TFBS or mutation, highly-expressed genes appear to have a high GC-skew around the TSS. As a GC-skew was also detected in the upstream regions of the TIS in fungal genes, this feature might be generated by a common mechanism in both groups. However, additional sequence data, and investigations into the patterns of nucleotide substitutions and their rates around the TSS, both between and within groups, will be necessary to elucidate the origin and mechanism of GC-skew around the TSS of plant and fungal genes.

**Figure 6:**
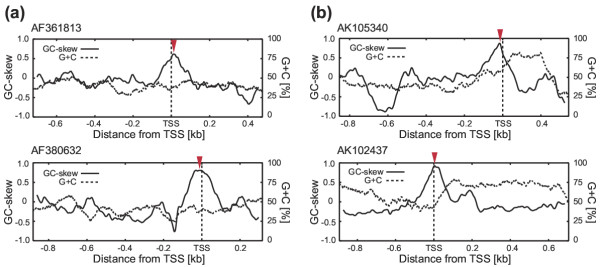
**Representative cases of GC-skew peaks located in the TSS of plant genes. **These figures show typical examples of GC-skew peaks located near the TSS in *Arabidopsis *(**a**) and rice (**b**). Red solid triangles represent the positions of the GC-skew peak. The corresponding GenBank entry IDs are: AF361813, AF380632, AK105340 and AK102437.

TSS prediction by the GC-skew peak was validated through a stepwise increase of cut-off values which demonstrated that the GC-skew could contribute to TSS prediction. Although the optimal GC-skew cut-off value depends on the specific situation, our results will be helpful in determining the optimal cut-off. Figure 6 shows representative cases in which the GC-skew peaks are located near the TSS in *Arabidopsis *and rice genes. The single index presented in this paper might not be sufficient to achieve accurate TSS prediction. However, our results indicate that GC-skew is a good candidate index for the TSS, promoter or first exon in plants. Thus, the combined use of GC-skew and other indices, or the incorporation of this index into pre-existing programs appears to be a realistic and effective approach for TSS prediction.

## Conclusion

Significant GC-skew (C > G) around the TSS is strictly conserved among monocot and eudicot plants (that is, angiosperms), and a similar skew is also seen in some fungi. The mean GC-skew at the TSS in the highly expressed genes was greater than that in the group with low expression. We therefore propose that the GC-skew around the TSS in some species of plants and fungi is associated with transcriptional activity. This is probably a result of DNA mutations during transcription initiation or the frequent use of strand-specific TFBS. Our findings also confirm that GC-skew has the potential to assist TSS prediction in plant genomes, where there is a lack of correlation among CpG islands and genes.

## Methods

### Data sources

Data for 13,095 full-length cDNAs from *Arabidopsis thaliana *were downloaded from The Institute for Genomic Research (TIGR) [23] (as of March 2, 2001) and the RIKEN *Arabidopsis *Genome Encyclopedia [24] (as of May 9, 2002). Genomic sequences for *Arabidopsis *were downloaded from The National Center for Biotechnology Information (NCBI) [25] (as of January 31, 2003). ORF information for *Arabidopsis *genes was obtained from the NCBI Entrez database [26], based on each of the original sequence IDs. For rice (*O. sativa *ssp. *japonica *c.v. Nipponbare), 28,469 full-length cDNA sequences [27] were retrieved from the Knowledge-Based *Oryza *Molecular Biological Encyclopedia (KOME) [28] with ORF information, and the genomic sequences were obtained from the Rice Genome Research Program [29] (as of October 16, 2002) and Syngenta Biotechnology Inc. (SBI) [30]. For analysis of human (*Homo sapiens*) DNA, 21,245 full-length cDNAs and genomic sequences were downloaded from the DNA Data Bank of Japan (DDBJ) [31] and Ensembl (Ver. 9.30) [32], respectively. Data for 9,872 full-length cDNA sequences (as of July 17, 2002) and genomic sequences of *Drosophila melanogaster *were obtained from the Berkeley *Drosophila *Genome Project [33,34]. Fungal genomic sequence data, including ORF information, were downloaded from the NCBI [35,36] for *S. cerevisiae *and *S. pombe*, and from the Fungal Genome Initiative (FGI) [37] for *A. nidulans*, *F. graminearum*, *M. grisea*, and *N. crassa*. Virtually assembled transcripts from 10 animal species, nine plant species, six species of fungi and 11 protist species were retrieved from the TIGR Gene Indices (TGI) [16,17].

### Mapping of cDNA to the genomic sequences

Redundant cDNA sequences showing at least 95% similarity in at least 95% of the regions, compared with other sequences were excluded from the full-length cDNA dataset for four species – *Arabidopsis*, rice, human and *Drosophila*. They were identified using BLASTN homology searching and CAP3 [38]. Also, any poly(A) tracts at the 3' -end of the cDNA sequences were eliminated. Finally, by mapping the cDNA sequences for corresponding genomic sequences using the BLASTN program and SIM4 [39], sequences both up- and downstream of the TSS were determined.

### Gene-expression data

SAGE data for *Arabidopsis *(10-day-old seedlings) [18] were used to examine the relationship between GC-skew in the TSS and gene-expression levels. In assigning the SAGE tags to genes, only data that showed one-to-one correspondence between the genes and tags were included. Genes with <100 counts per million were defined as the low-expression group (504 genes) and those with >100 per million the high-expression group (689 genes). The mean GC-skew values in a 100-bp window at the TSS were calculated for both groups. Genes with a G+C content at the TSS of <0.3 were eliminated from the dataset in advance.

### GC-skew peak detection

As an initial step towards detecting the GC-skew peaks, GC-skew values at each position in the sequences were calculated using the sliding-window technique (window size = 100 bp; shift size = 1 bp). Next, we attempted to smooth the spectrum, to reduce the noise and to simultaneously determine the peaks, using the S-G filter [19]. The first-order derivative of the smoothed GC-skew value *g*_*i *_at position *i *is given by the following equation:



Here, *f*_*i*+*n *_is the original GC-skew value in the position *i *+ *n*. *n*_*l *_and *n*_*r *_represent the lengths of the filter window to the left and right of position *i*, and were set to 20 in this analysis. *C*_*n *_denotes the set of weight coefficients and corresponds to the first-order derivatives of the quartic polynomial. The position of the GC-skew peak *i *was defined as the zero-crossing point, which is the point satisfying the following condition:

*g*_*i*_·*g*_*i* + 1_ ≤ 0 ∩ *g*_*i* + 1_ < 0

The peak was only counted as a TSS candidate if a GC-skew value at that peak satisfied the particular cut-off value, and the G+C content was ≥ 0.3 in the window. When more than one peak occurred within 50 bp, they were regarded as identical and the peak with the highest GC-skew value was accepted. Using this procedure, TSS prediction was conducted with stepwise changes in the cut-off values of the GC-skew (-0.9 to 0.9) at the peak.

The predictive accuracy was verified by counting TSS candidates located within 100-bp up- and downstream of the actual TSS as true positives (*TP*). Where more than two TSS candidates coexisted within 100-bp either up- or downstream of the TSS, they were counted as one TSS candidate. The rest of the candidates, which were located in inappropriate regions, were counted as false positives (*FP*). To compare results for different cut-offs, the correlation coefficient (*φ*), which is one of the measures used to compare predictive performance [40], was calculated as follows:



Here, *TN *and *FN *denote the number of true and false negatives, respectively. In this analysis, the total number of negatives (*N*) was defined as the maximum number of possible TSS candidates in the non-TSS regions. Thus, *TN *was calculated as *N*-*FP*.

### Correlation coefficient between the two nucleotide frequencies

The correlation coefficient *r*_*ij*_(*p*) between nucleotide *i *and *j *at a position *p *up- and downstream of the TSS was defined as follows:



Here, *i*_*k*_(*p*) and *j*_*k*_(*p*) are the frequencies of *i *and *j *at a position *p *in the sequence *k*, respectively.  and  are the mean frequencies of *i *and *j *at *p*. The nucleotide frequency in each position was the number of each nucleotide in a 100-bp window.

## Authors' contributions

SF carried out the computational and statistical analysis, and drafted the manuscript. TW and MT participated in the design and coordination of the study. All of the authors have read and approved the final manuscript.

## Supplementary Material

Additional File 1 This file contains three figures: S1, showing the nucleotide frequency around the TSS; S2, showing the AT/GC-skew in both up- and downstream regions of the TSS in plants; and S3, showing the correlation between the two nucleotide frequencies around the TSS in human and *Drosophila*. Click here for file
