# Supplementary material for: GC-compositional strand bias around transcription start sites in plants and fungi
Source: BMC Genomics. 2005 Feb 28;6:26. doi: 10.1186/1471-2164-6-26 (PMC555766; doi:10.1186/1471-2164-6-26)
Supplement: Additional File 1 — This file contains three figures: S1, showing the nucleotide frequency around the TSS; S2, showing the AT/GC-skew in both up- and downstream regions of the TSS in plants; and S3, showing the correlation between the two nucleotide frequencies around the TSS in human and Drosophila. [file 1471-2164-6-26-S1.pdf]

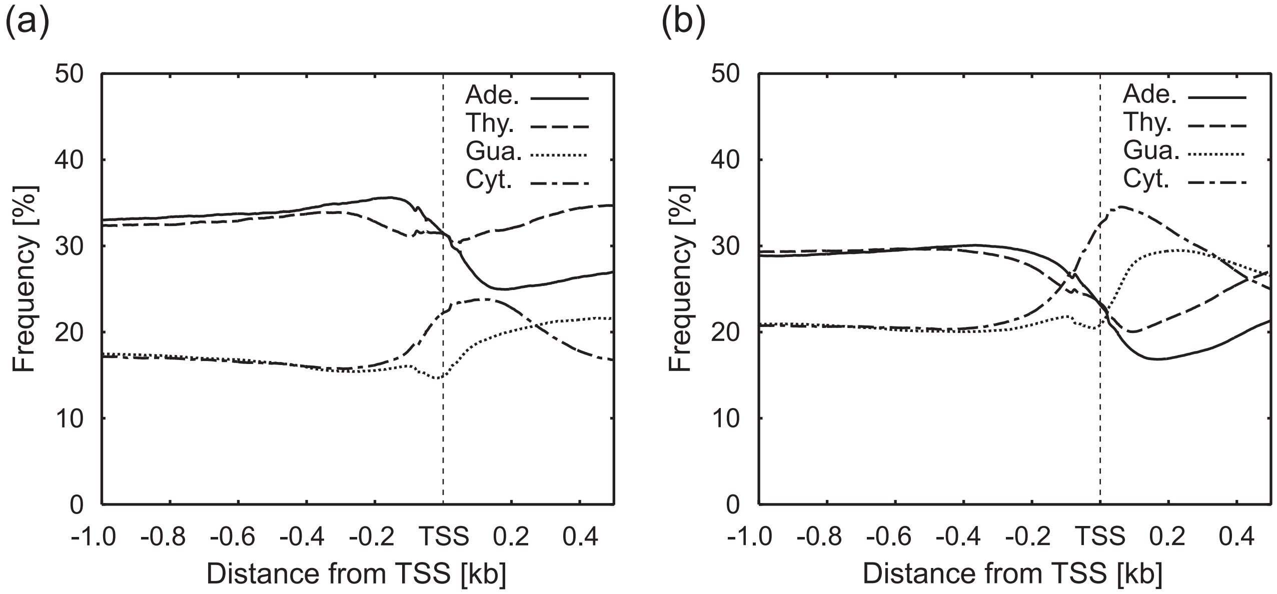

Figure S1 - Nucleotides frequency around the TSS. Mean values of each nucleotide frequency were calculated taking advantage of the sliding window technique (window size = 100 bp; shift size = 1 bp) in (a) *Arabidopsis* and (b) rice. The same datasets as those of the calculation of GC-skew values were used.

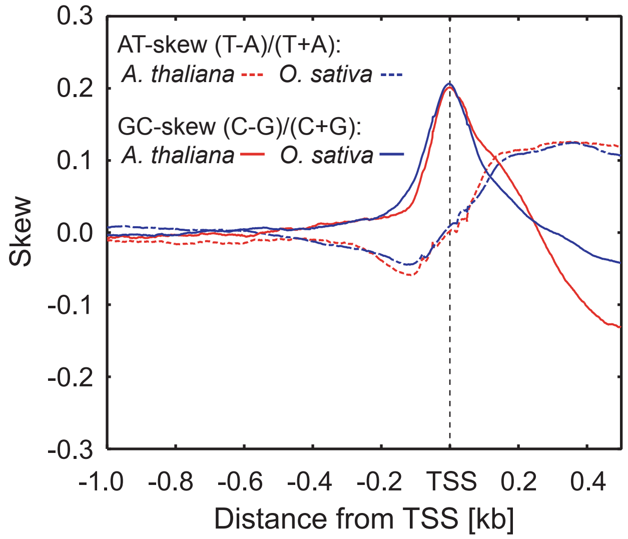

Figure S2 - AT/GC-skew in up-/downstream regions of the TSS in plants. Up-/downstream regions of the TSS, 1.0-kb and 0.5-kb in length, respectively, were analyzed in datasets from two plants, 7,708 loci for *Arabidopsis*, and 14,868 loci for rice. The graph shows the mean AT-skew (=(T-A)/(T+A)) and GC-skew (=(C-G)/(C+G)) values that were calculated for sequences of two plants, using the sliding window technique (window size = 100 bp; shift size = 1 bp).

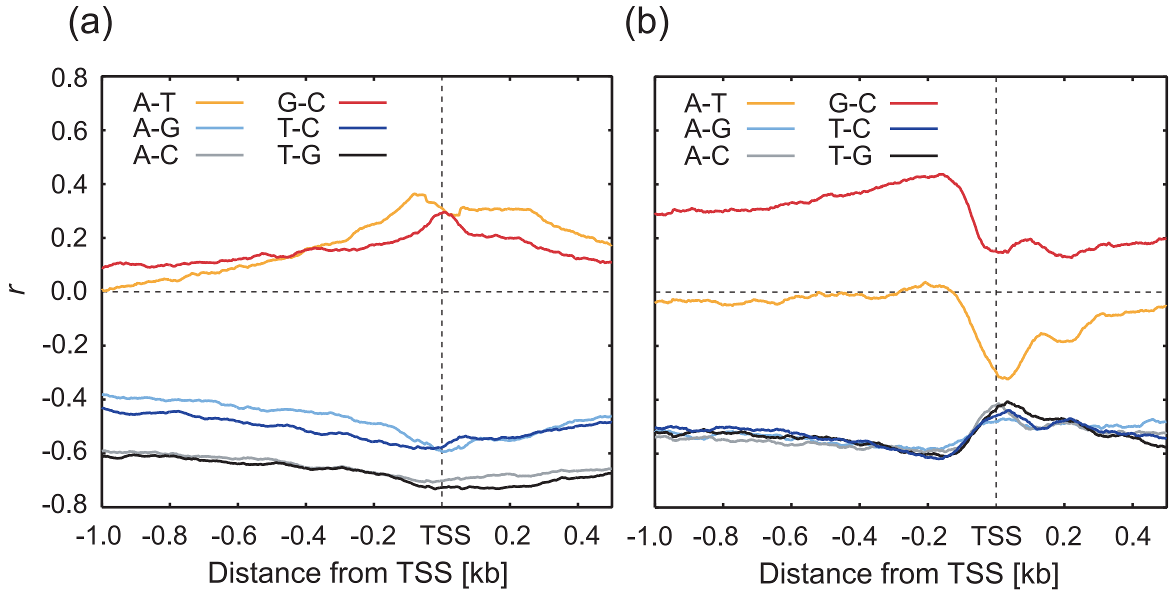

Figure S3 - Correlation between the two nucleotide frequencies around TSS in human and *Drosophila*. The figure illustrates the correlation coefficients (r) between the two nucleotide frequencies, A-T, A-G, A-C, G-C, T-C, and T-G, at each point around TSS of (a) human, and (b) *Drosophila* genes. Each nucleotide frequency at a particular position was defined as the frequency of the nucleotide in a 100-bp window at that position. Correlation coefficients were calculated using these frequencies (see Methods section). The numbers of sequences analyzed were 14,053 for human and 8,344 for *Drosophila*.
